# A vocal cord palsy caused by the uterine cancer metastatic tumor in the mediastinum revealed in a patient with a thyroid lesion: a case report and review of the literature

**DOI:** 10.1186/s13256-024-04461-y

**Published:** 2024-04-04

**Authors:** Andrii Hryshchyshyn, Andrii Bahrii, Andrii Bazyshen, Hryhorii Bohush

**Affiliations:** 1 Medical Center Hormony, Vinnytsia, Ukraine; 2 Kyiv City Endocrinological Center, Kyiv, Ukraine; 3 National Pirogov Memorial Medical University, Vinnytsia, Ukraine

**Keywords:** Thyroid surgery, Mediastinum tumor, RLN palsy, Recurrent laryngeal nerve paresis, VCP, Vocal cord paralysis, Uterine cancer, Metastasis, Vocal fold palsy, Endometrial clear cell carcinoma

## Abstract

**Background:**

The main cause of vocal cord palsy (VCP) is idiopathic impairment of the recurrent laryngeal nerve (RLN). However, solid tumors along the pathway of the RLN can also impact the nerve’s function. We presented a patient with a thyroid lesion and VCP due to a bulky metastatic mass (uterine cancer) on the aortic arch field in the mediastinum. The report aims to show the significance of comorbid tumors in thyroid pathology and the importance of additional diagnostic methods in avoiding unnecessary surgeries. A patient’s lifetime and the outcome of the disease were also presented.

**Case presentation:**

A 58-year-old Ukrainian woman with a hoarse voice, intermittent dry cough, and weakness was presented to an endocrine surgeon. Thyroid pathology included signs of hypothyroidism treated with Thyroxine 112.5 µg and a nodule in the left lobe. The lesion is located on the posterior aspect of the lobe, which could probably be a cause of RLN involvement. Fine needle aspiration biopsy (FNAB) was performed twice with Bethesda category 2 result. Fibrolaryngoscopy (FLS) revealed the median position of the left vocal cord. Idiopathic, laryngeal, and thyroid causes of the VCP were excluded. Additionally, the patient displayed her anamnesis of the endometrial clear cell carcinoma following hysterectomy, external beam radiation therapy, and chemotherapy. The mediastinal metastasis was revealed sixteen years later. A chest computed tomography (CT) with intravenous contrast was done. A bulky tumor was found right under the aortic arch. Subsequently, the voice complaints reduced significantly after 4 chemotherapy courses. Cancer progression had led to the appearance of lymph node metastases on the supraclavicular region. Following six months the 60-year-old patient had passed away.

**Conclusion:**

A history of the disease should always be kept in mind when assessing a patient’s complaints. VCP in case of thyroid pathology and previous secondary malignancy may be caused by metastatic tumor anywhere along the RLN pathway. Such a rare case shows the importance of additional methods of examination which may avoid unnecessary thyroid surgeries.

## Introduction

Vocal cord palsy (VCP) is a mobility impairment of the vocal ligaments [1]. Surgical procedures are the second cause of VCP (37%) followed by secondary tumors and lesions (63%), which do not originate from the RLN [2]. A long course of both RLNs should be kept in mind when the cause of a paresis tries to be found. The left RLN divides in the middle mediastinum behind the aortic arch, loops there, and passes cranially to the voice box through the neck [3].

cknowledgements of a pathway of the RLN in the absence of neck surgeries should be taken into account.

Endometrial clear cell carcinoma (ECCC) is a rare uterine cancer pathology with about 9% occurrence [4]. Up to 39% of all ECCC metastases are found in the lungs, but no mentions of VCP associated with uterine cancer were presented [5]. Myssiorek noticed in his article, that nonlaryngeal tumors consisted of 17–32% of RLN dysfunction [6]. Endotracheal intubation in 0.1% of this manipulation causes vocal cord palsy, which is known to be very rare [7]. In relatively frequent cases, up to 62% of all VCPs could be explained as idiopathic [8]. Song *et al.* noticed only 2,6% of lesions with vocal fold immobility which were confirmed as mediastinal masses. None of them originated from gynecological pathology [9]. Also, left RLN impairments were encountered in the vast majority of cases with a rate of 63%.

Additional imaging methods may significantly improve a differential diagnosis. For instance, an X-ray of the chest cannot detect small mediastinal lesions. Therefore, a CT scan is considered the most important in evaluating the mediastinal structures and masses [10]. Fibrolaryngoscopy (FLS) is the gold standard for the detection of VCP [11].

**Fig. 1:**
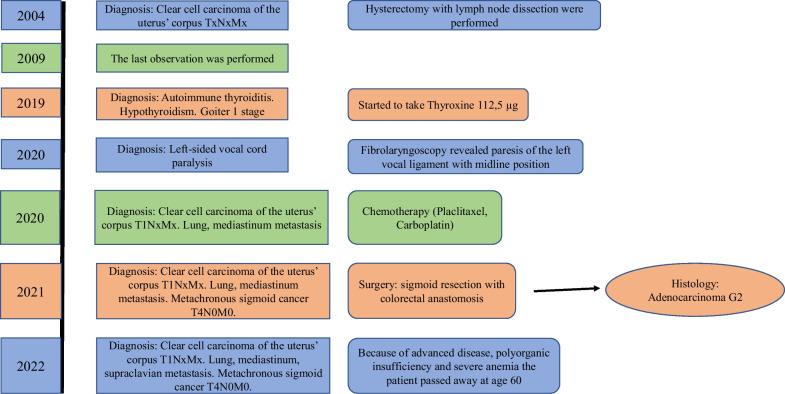
A detailed lifetime of the patient

We thoroughly evaluated the case history of our patient to show the key objectives (Fig. 1). The diagnostic methods, the importance of anamnesis, and differential diagnosis were presented in this case report about VCP.

## Case presentation

A Ukrainian woman, aged 58, was presented with hoarseness and cough complaints. She had suffered from breathing problems during the last 10 months. Autoimmune thyroiditis with a nodule in the left lobe had been treated with Thyroxine 112.5 µg since 2019. No other masses on the chest X-ray were found. The patient mentioned a history of endometrial clear cell carcinoma (ECCC), which had been treated 16 years before. She underwent a total hysterectomy with lymph node dissection. Subsequently, several courses of chemotherapy and external beam radiation therapy were performed. The patient had postponed observational procedures within five years after the surgery. She did not make an appointment with an oncologist for the next six years as well. The presence of hoarseness 16 years after the hysterectomy forced the woman to be observed by the surgeon.﻿

## Clinical findings

**Fig. 2:**
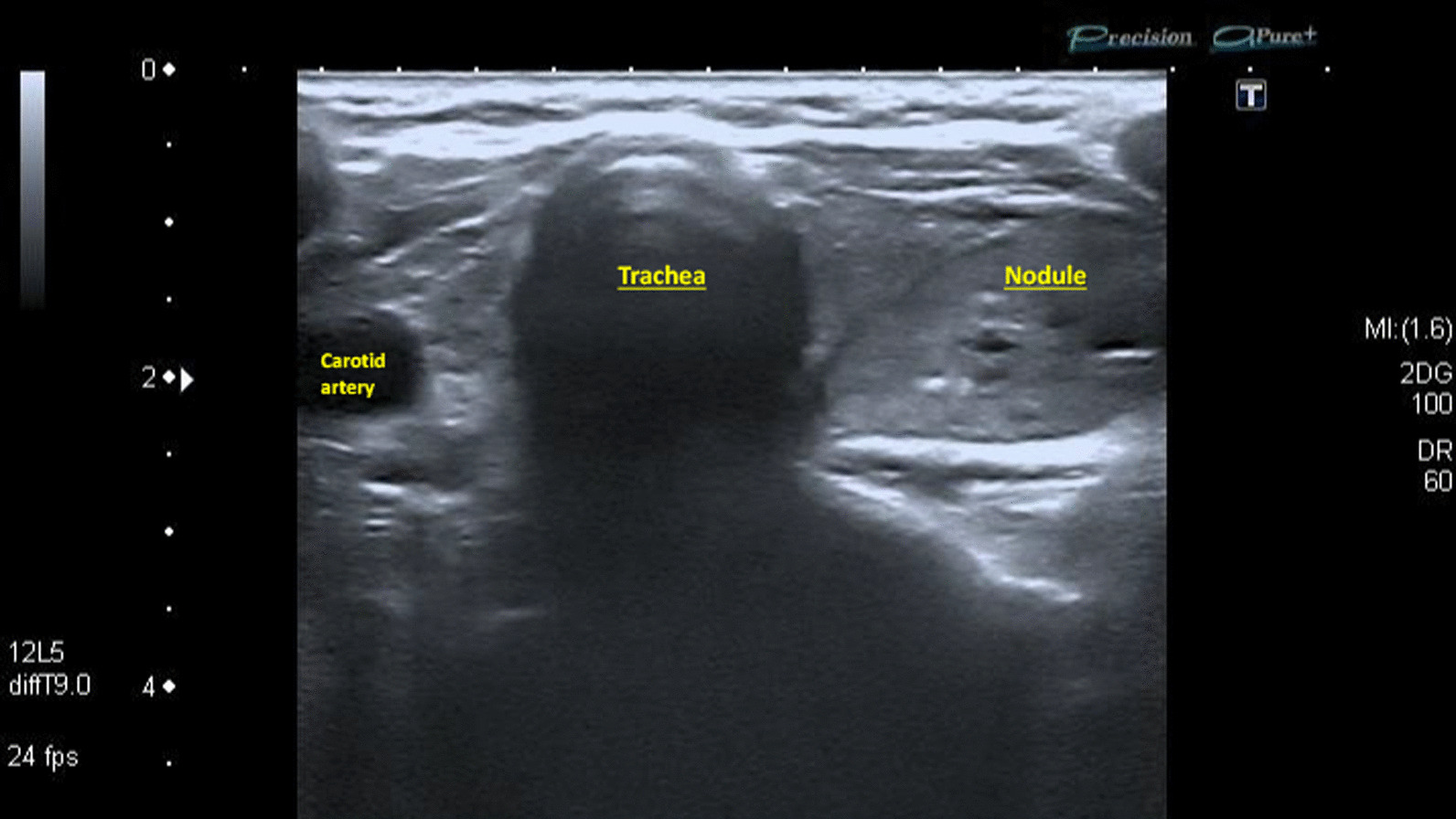
The US image of the thyroid gland in the patient with a nodule. Severely hypoechoic thyroid parenchyma is with a nodule in the left lobe. The lesion is hypoechoic, with regular margins and hyperechoic inclusions. The medial border of the nodule is close to the trachea and virtual course of the left  recurrent laryngeal nerve

**Fig. 3:**
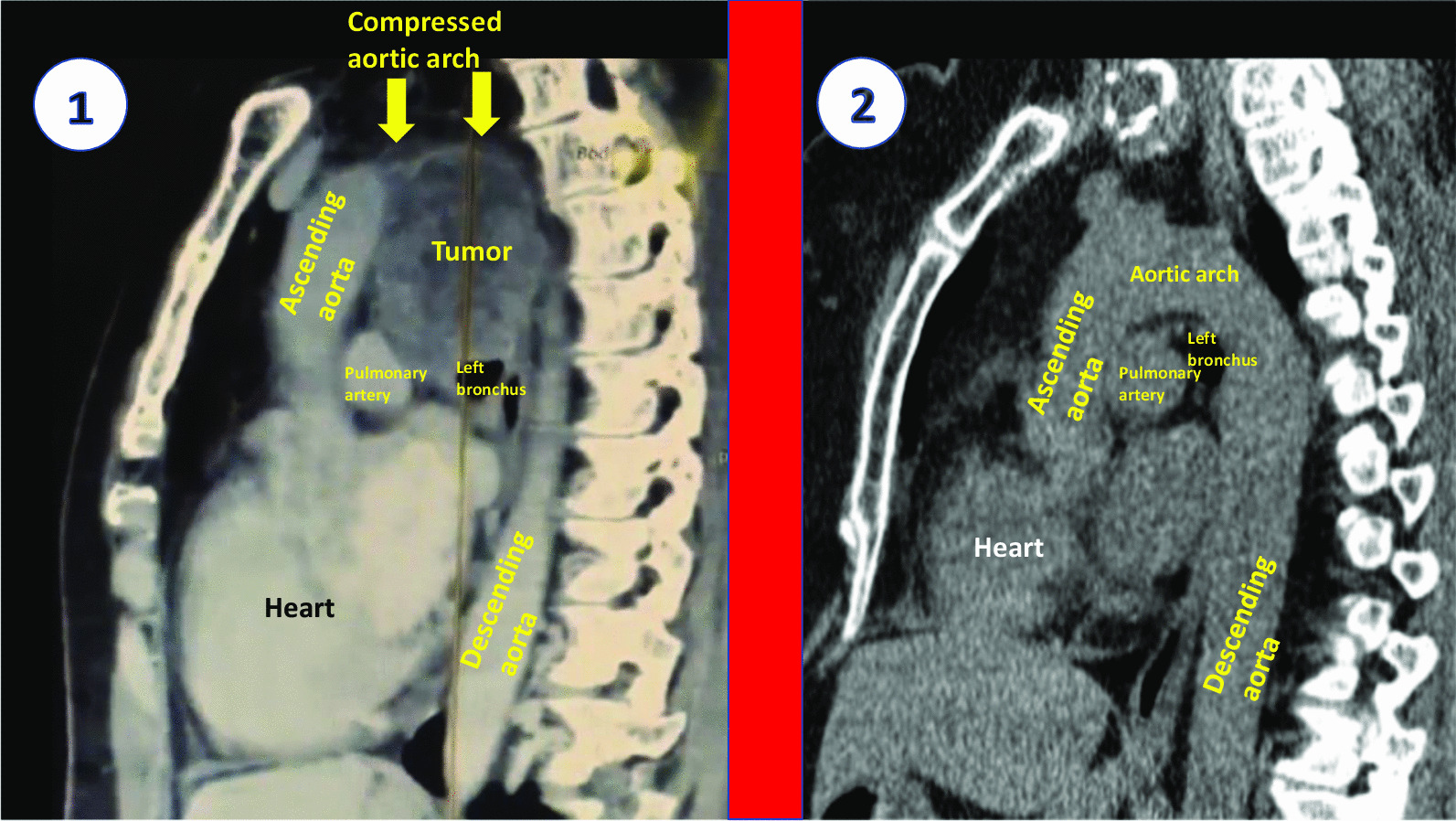
The chest computed tomography scan with a tumor of the mediastinum (1) and without pathology (2). The metastatic mass is circle-shaped, 77*43*65 mm, and heteroechoic. The tumor tightly abuts the aortic arch right by the course of the left recurrent laryngeal nerve.  Computed tomography scan of a different patient without any pathology of the mediastinum. The image is divided by a wide red line into two parts. All the important structures are marked with yellow, white and black inscriptions

The main complaints of the patient were hoarseness, dry permanent cough, and severe fatigue, which had appeared 10 months earlier. According to the history of thyroiditis, the neck US was performed (Fig. 2). Hypoechoic, heterogenic parenchyma with hypoechoic, regular contours, low central vascularity, 32 × 14 mm oval-shaped nodule in the left lobe were found. The lesion had microcalcification, located on the posterior aspect of the lobe close to the trachea and RLN passing. The patient underwent fine needle aspiration biopsy (FNAB) with a Bethesda category 2 result. FLS was done, the left vocal fold bowing and fixed in the median position. Also, no additional masses in the larynx were detected. VCP was considered to be idiopathic; however, a history of uterine cancer forced to recommend the chest CT scan. On the images, the huge additional mass in the middle mediastinum was identified. It had irregular borders, 77 × 65 mm diameter, hypoechoic and monogenic structure without calcifications. This lesion abutted to the posterior wall of the aortic arch and significantly compressed a lumen of the vessel (Fig. 3). The patient was referred to the tertiary hospital and metachronous sigmoid cancer was diagnosed. Initially, 6 courses of chemotherapy (Paclitaxel, Carboplatin) due to uterine cancer metastasis to the left lung and mediastinum were done. It was noted that hoarseness significantly facilitated after the tumor size decreased during treatment. After chemotherapy, sigmoid resection with lymph node dissection and primary colorectal anastomosis were performed at the hospital. Subsequently, 7 courses of chemotherapy (Paclitaxel, Carboplatin, Avastin) against colon cancer were performed.

During the last several years, TSH level was in the range of 2.5–3.9 mU/L (reference 0.45–4.0) with Thyroxine 112.5 µg supplementation.

During one and a half years following the last colon surgery, the patient had been undergoing palliative therapy. Two years after the first signs of metastatic masses to the mediastinum the woman passed away at age 60 due to cancer progression and polyorganic insufficiency.

The CARE guidelines were applied in writing this report.

## Discussion

Abeler *et al.* described the metastatic patterns of ECCC. Among 50 patients with distant metastases, uterine cancer masses were identified in the lungs in about 39% of patients [5]. It is considered that ECCC metastasis in the lung and mediastinum, which causes VCP, hasn’t been published to date. Our case thoroughly describes all symptoms, history, and consequences of this rare pathology.

Ortner’s syndrome—is a state of VCP in patients with any relation to cardiovascular diseases. It includes left atrial enlargement, pulmonary hypertension, aortic anomalies, aneurysm of the right subclavian artery, and ductus arteriosus [12]. Alexandra Mesquita (2022) presented a case of left-sided VCP as a result of the aortic arch aneurysm in a 60-year-old woman [13]. A single sign of this state was hoarseness. The patient had been treated with aortic arch replacement, subsequently, the aneurysm volume was reduced significantly. However, VCP did not disappear due to long-time stretching and compression of the RLN. The voice of our patient was slightly improved after the fourth course of chemotherapy. Presumably, it could have happened because of a relatively short period of compression and the absence of invasion into the RLN.

For instance, computed tomography with intravenous contrast can identify any lesions or tumors within the RLN pathway. This particular method is highly helpful in distinguishing an enormous metastatic mass in a mediastinum [14]. A CT scan of the neck and vocal cord can also shed light on the location of important structures of the larynx and supraglottic space. Si Wei Kheok (2021) noticed features of VCP which were as follows: the dilatation of the ipsilateral piriform sinus, medialization of the aryepiglottic fold and enlargement of the ipsilateral laryngeal ventricle, anteromedial deviation of the arytenoid cartilage [15].

Ultrasound (US) is the gold standard of a non-invasive thyroid lesion examination [16]. Our patient had signs of inflammation of the thyroid parenchyma, a lesion in the left lobe. This nodule was heteroechoic with micro- and macrocalcification, which was supposed to be suspicious. Also, the most important medial aspect of the lesion was close to the dangerous zone of the left RLN path and trachea. Therefore, the patient would have to perform a CT scan of the chest and neck to identify the signs and source of VCP in the larynx and middle mediastinum, respectively. Radiological methods should be used in difficult cases to understand the cause of the pathology.

The other cause of RLN palsy is the endovascular treatment of the aortic arch hypoplasia. Implantation of a composite stent into the lumen of the artery leads to significant enlargement of the vessel’s volume. The RLN passes directly underneath the aortic arch. Subsequent nerve stretching caused left RLN palsy, which was recovered in advance [17]. Also, the other endovascular surgery with aortic stent-graft placement can entail left VCP. An extra dilatation of the vessel is considered to be a predictor of RLN paresis [18]. These reports represented an iatrogenic injury of the RLN, which differs from our case. However, it led to a similar complication, that should be identified and treated. In the above-mentioned case, the nerve was stretched significantly because of the additional vessel’s dilatation. As opposed to that, the patient with a mediastinum tumor had nerve compression due to the permanent growth of the metastases. The treatment is different in these cases.

It seems to be found no articles in the published literature about ECCC metastasis which caused VCP. The limitations of our case were as follows: reference to the tertiary hospital without feedback, retrospective collection of data of the case history, doctors in the tertiary hospital were not focused on VCP and didn’t perform FLS to evaluate vocal ligament mobility, lack of additional data about the surgery, the volume of the lymph node dissection, initial chemo- and radiotherapies.

## Conclusion

Vocal cord palsy is an extremely rare complication of ECCC metastasis. Secondary malignancies in anamnesis of a patient should be taken into account in searching for a cause of VCP. Thyroid lesions should be thoroughly evaluated in case of malignancy suspicion and RLN invasion. Supplemental investigational methods (a CT scan, neck US, and FLS) should be used in all equivocal processes. A correct diagnosis needs to be defined to avoid unnecessary surgeries. Non-conventional cases of rare and common pathology should be certainly published and shared to help other specialists in their practice.

## Data Availability

The author will provide all the data on request.

## Abbreviations

VCP: Vocal cord palsy
RLN: Recurrent laryngeal nerve
FNAB: Fine needle aspiration biopsy
FLS: Fibrolaryngoscopy
CT: Computed tomography
US: Ultrasound
ECCC: Endometrial clear cell carcinoma
CARE: CAse REport guidelines
